# Comparison of antibody response following the second dose of SARS-CoV-2 mRNA vaccine in elderly patients with late-stage chronic kidney disease

**DOI:** 10.1186/s41100-022-00402-x

**Published:** 2022-04-05

**Authors:** Masatoshi Matsunami, Tomo Suzuki, Junko Fukuda, Toshiki Terao, Kohei Ukai, Shinnosuke Sugihara, Takumi Toishi, Kanako Nagaoka, Mayumi Nakata, Mamiko Ohara, Jun Yashima, Hiroshi Kuji, Kosei Matsue

**Affiliations:** 1 Department of Nephrology, Kameda Medical Center, 929 Higashi-cho, Kamogawa, Chiba 296-8602 Japan; 2 Renal Transplant Center, Kameda Medical Center, Chiba, Japan; 3 Division of Hematology/Oncology, Department of Internal Medicine, Kameda Medical Center, Chiba, Japan; 4 Department of Urology, Kameda Medical Center, Chiba, Japan

**Keywords:** COVID-19, SARS-CoV-2 IgG antibody, Vaccination, Chronic kidney disease, Hemodialysis, Elderly patients

## Abstract

**Background:**

Currently, it is unclear whether the progression of chronic kidney disease (CKD) could be an independent predictor of antibody response after administration of a COVID-19 vaccine. This study aimed to investigate the immune response to COVID-19 vaccination in patients with CKD stage G4 to G5 without renal replacement therapy and G5D using the recommended dose and schedule.

**Methods:**

This retrospective single-center cohort study evaluated immunogenicity regarding antibody response after COVID-19 vaccination in our hospital for late-stage CKD patients aged ≥ 60 years. We evaluated antibody responses in 48 patients with CKD G4, 35 patients with CKD G5, and 70 patients undergoing hemodialysis (HD; CKD G5D).

**Results:**

After the second vaccination, anti-SARS-CoV-2-S (Spike) IgG levels were found to be positive (> 0.8 U/mL) in all CKD G4 and G5 patients (100%), and 69 of 70 HD patients (98.5%). The median (interquartile range [IQR] S-IgG level (Ab titers) was 358 [130.2–639.2], 218 [117–377], and 185.5 [95.1–323.5] U/mL in the CKD G4, G5, and HD groups, respectively. The median S-IgG levels were significantly lower in the HD group than in the CKD G4 group (*p* < 0.01). However, there was no significant difference in the antibody titers between the CKD G4 and G5 groups. To further analyze the decline in S-IgG levels after 6 months, we additionally assessed and compared antibody titers at 1 month and 6 months after the second vaccination in the HD group. Compared with the median S-IgG levels of 185.5 [95.1–323.5] U/mL 1 month after the second dose, the median S-IgG level 6 months thereafter was significantly decreased at 97.4 [62.5–205.5] U/mL (*p* < 0.05).

**Conclusions:**

We highlight two major factors of variability in the vaccine response. First, in elderly patients with late-stage CKD, antibody titers tended to be lower in the G5D group than in the G4 and G5 groups despite the shorter time since vaccination; therefore, CKD stage progression might cause a decline in antibody titers. Second, waning immune responses were observed 6 months after second dose administration in HD patients advocating a potential need for a third booster dose vaccine after 6 months.

## Background

The high-risk groups for coronavirus disease 2019 (COVID-19)-mediated critical illness include those with obesity, diabetes mellitus, older age and chronic kidney disease (CKD) [1–3], with CKD emerging as the most prevalent of all [3]. Thus, patients with CKD, with their elevated risk of mortality from COVID-19, require urgent action for preventing severe outcomes.

Presently, studies have shown that responses to COVID-19 mRNA vaccines are likely to be lower in patients with CKD than in the general population [4–6]. Therefore, immunization against  COVID-19 with effective vaccines is an important component of health-maintenance strategies for these patients [4].

Currently, it is unclear whether the progression of CKD could be an independent predictor of antibody response after administration of a COVID-19 vaccine. This study aimed to investigate the immune response to COVID-19 vaccination in patients with CKD stage G4 to G5 without renal replacement therapy (RRT) and G5D using the recommended dose and schedule.

## Methods

### Study design and participants

This retrospective single-center cohort study was performed in Kameda Medical Center to evaluate immunogenicity in terms of antibody response after COVID-19 mRNA vaccination in patients with late-stage CKD aged 60 years or older.

Due to Japan’s vaccine delivery systems, group vaccination was conducted mostly with two doses of Comirnaty COVID-19 vaccine (BioNTech-Pfizer BNT162b2).

We evaluated antibody responses in 48 patients with CKD G4, 35 patients with CKD G5, and 70 patients undergoing hemodialysis (HD) (CKD G5D). We excluded patients aged below 60 years, receiving treatment for immunosuppression, and with malignancy or hematologic disorders.

As we reported earlier [7], severe acute respiratory syndrome coronavirus 2 (SARS-CoV-2)-specific antibodies in HD groups were evaluated and compared to that of control group; thus, in this study, we evaluated the antibody responses among late-stage CKD patients based on a residual renal function. The details of the control group, which is important for this study, are shown in Table 1 because these data were not presented in our previous report. The control group was composed of 35 participants (a population expected to have optimal antibody response) who were volunteers that met the criteria of over 60 years of age with no evidence of kidney failure, active cancer or ongoing treatment for immunosuppression. They were selected over a four-month period by consecutive sampling from patients visiting our gastroenterology outpatient clinic.

**Table 1:** Comparison of different variables between study groups

Variables	Control n = 35	CKD stage G4 n = 48	CKD stage G5 n = 35	CKD stage G5D n = 70
Age (years)	75.1 ± 6.5	77.1 ± 9.0	75.3 ± 8.9	73.1 ± 8.5
Age > 60 years	100%	100%	100%	100%
Male sex	42.8%	70.8%	65.7%	67.1%
Diabetes mellitus	0%	41.6	40.0%	38.5%
Serum creatinine (mg/dL)	0.75 ± 0.17	2.27 ± 0.55	4.80 ± 1.51	9.35 ± 2.23
eGFR (mL/min/1.73 m^2^)	67.6 ± 12.9	22.0 ± 4.9	9.8 ± 2.5	4.7 ± 1.2
Antibody titers (U/mL)	533 (275.5–1100)	358 (130.2–639.2)	218 (117–377)	185.5 (95.1–323.5)
Antibody positivity	100%	100%	100%	98.5%
Sample taken after second vaccination (days)	33 (23–45)	100 (79–127.5)	97 (84.7–110)	43 (36–50)

Values are expressed as mean ± SD for continuous variables with normal distribution and as median rank (25th-75th percentiles) for continuous variables without normal distribution

All participants had received the first and second dose of COVID-19 mRNA vaccines (following the recommended interval of 21 days for the BNT162b2 vaccine) between May 12, 2021 and October 6, 2021. Sample collection on antibody titers follow-up was continued until November 24, 2021.

### Humoral response assessment

Serum samples were tested for SARS-CoV-2 antibodies (immunoglobulin G [IgG] levels) using the Elecsys® Anti-SARS-CoV-2 S RUO (Roche Diagnostics, Basel, Switzerland) test system. Antibody titers > 0.8 U/mL were considered as positive immune response to vaccination.

### Outcomes

The primary outcomes evaluated in this study included quantitative humoral responses to the second dose of COVID-19 mRNA vaccine. Anti-SARS-CoV-2-S (Spike) IgG levels were evaluated among the CKD G4, G5, and G5D groups. In the G5D group, a comparison between 1 and 6 months after the second vaccination was also performed.

### Statistical analysis

The data were analyzed using GraphPad Prism 7.0 (GraphPad Software, San Diego, CA). To compare the three groups (non-normally distributed samples), the data were analyzed using the non-parametric Kruskal–Wallis and post hoc Dunn’s tests. Mann–Whitney U test was used to compare non-normally distributed data between two groups. *p < 0.05 and **p < 0.01 are depicted.

### Ethical considerations and disclosures

All patients provided written informed consent to participate in this study, which was approved by the institute’s committee on human research (Approval Number 21–025).

## Results

Table 1 lists and compares demographic and laboratory data of the study groups. CKD G4 consisted of 34 males (70.8%), with a mean age of 77.1 years, CKD G5 consisted of 23 males (65.7%), with a mean age of 75.3 years, and HD group consisted of 47 males (67.1%), with a mean age of 73.1 years. The median time on dialysis was 5.5 years in the HD patients. The percentage of male patients and the incidence of diabetes mellitus were almost the same in the G4, G5, and G5D groups.

After the second vaccination, anti-SARS-CoV-2-S (Spike) IgG levels were found to be positive (> 0.8 U/mL) in all CKD G4 and G5 patients (100%), and 69 of 70 HD patients (98.5%). The median (interquartile range [IQR]) S-IgG level (Ab titers) was 358 [130.2–639.2], 218 [117–377], and 185.5 [95.1–323.5] U/mL in the CKD G4, G5, and HD groups, respectively.

**Fig. 1:**
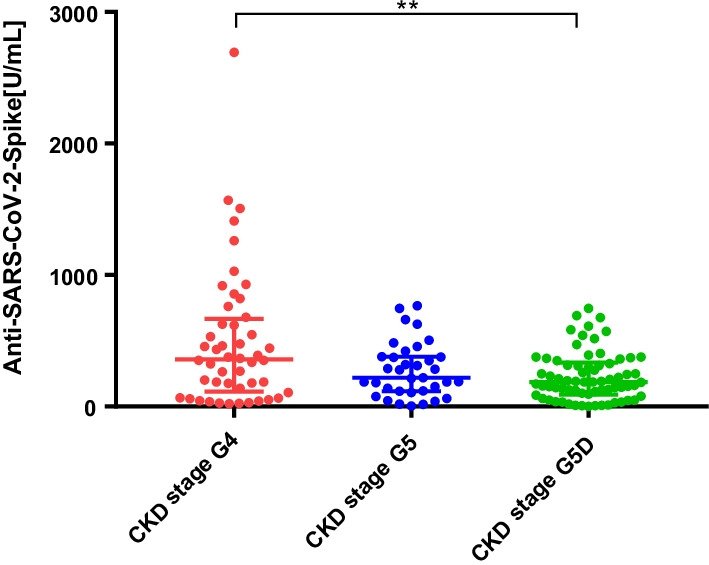
Antibody response following vaccination with the second dose of coronavirus disease 2019 (COVID-19) vaccine. The median antibody titers after second vaccination were significantly lower in the HD group than in the CKD G4 group (*p* < 0.01). There was no significant difference in the antibody titers between the CKD G4 and G5 groups

The median S-IgG levels were significantly lower in the HD group than in the CKD G4 group (*p* < 0.01) (Fig. 1).  However, there was no significant difference in the antibody titers between the CKD G4 and G5 groups (Fig. 1). No life-threatening allergic reaction or side effect was observed post-vaccination.

**Fig. 2:**
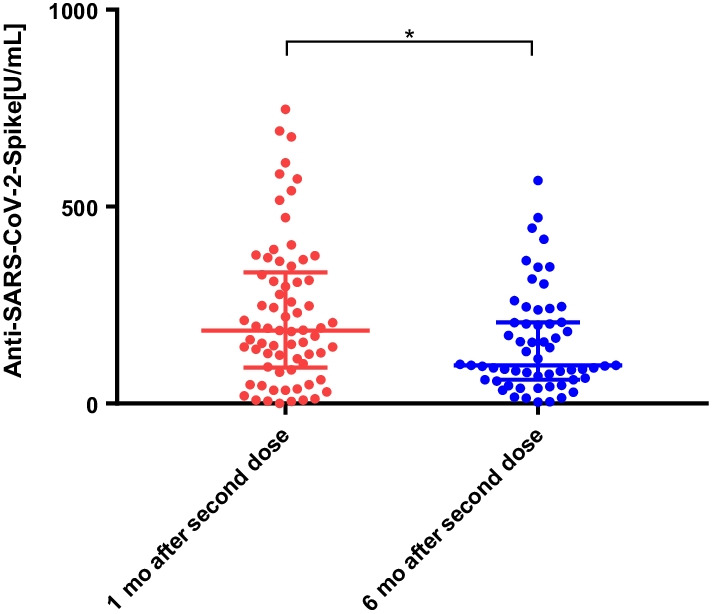
Antibody response following vaccination with the second dose of coronavirus disease 2019 (COVID-19) vaccine in hemodialysis patients. The median antibody titer decreased from 185.5 [95.1–323.5] U/mL 1 month after the second vaccination to 97.4 [62.5–205.5] U/mL 6 months after the second vaccination (*p* < 0.05)

To further analyze the decline in S-IgG levels after 6 months, we additionally assessed and compared the antibody titers at 1 month and 6 months after the second vaccination in the HD group. Compared with the median S-IgG level 1 month after the second dose (185.5 [95.1–323.5] U/mL), the median S-IgG level 6 months thereafter was significantly decreased (97.4 [62.5–205.5] U/mL) (*p* < 0.05) (Fig. 2).

## Discussion

We retrospectively evaluated the antibody responses of patients with CKD stages G4, G5, and G5D after the second-dose administration of the COVID-19 mRNA vaccine. It has been reported that obesity, diabetes mellitus, older age, male sex, and CKD affect response to COVID-19 vaccine [1–3]; however, in our study, the percentage of male patients and the incidence of diabetes mellitus were almost the same among the G4, G5, and G5D groups; thus, we evaluated the antibody responses based on a gradual loss of kidney function.

There is growing evidence for the protective efficacy of the COVID-19 vaccine [8–10]. However, due to their impaired immune system, patients with CKD and those undergoing RRT produce a suboptimal response to the vaccine [7]. Furthermore, there have been no clear data on the COVID-19 vaccination of patients with CKD stages G4 to G5.

In our study, median antibody titers in patients with GFR 15–29, < 15, and < 5 mL/min were 358, 218, and 185.5 U/mL, respectively. Thus, the degree of renal impairment may influence the titers of anti-SARS-CoV-2-S IgG. In later stages of CKD, especially in the hemodialysis stage, vaccination was more likely to induce a poor antibody response [11]. Therefore, kidney function appears to be an independent predictor of IgG levels.

Regarding the antibody titers 6 months after the second dose of COVID-19 vaccine, waning immune responses was observed in this study. These findings are consistent with previous preliminary reports [8, 12–14]. Moreover, these studies indicate that immune responses to vaccines are considerably reduced in patients undergoing dialysis, for whom a vaccination strategy including three doses of vaccine has been recommended [13], and administration of a third dose of the BNT162b2 vaccine to solid-organ transplant recipients significantly improved the immunogenicity of the vaccine [12]. Therefore, our results confirm the potential need for booster doses, especially in those with lower antibody titers, after administering the second dose of COVID-19 vaccine.

With regard to the third-dose vaccination strategy in patients with late-stage CKD and low immune responses, we have to consider the effective dose and schedule of the vaccine [13–15]. To increase effectiveness in CKD patients, higher vaccine doses or an increased number of vaccine injections may be necessary for booster vaccination, similar to influenza and hepatitis B vaccinations [16].

We encourage further studies to assess whether a stronger antibody response could be obtained with administration of higher doses of vaccine booster against COVID-19 in late-stage CKD.

The study limitations include variation in sampling  date among patients with CKD stages G4–5 and the lack of cellular immune response data. In the G4 and G5 groups, differences in the intervals and frequency of outpatient visits were observed, rendering it difficult to compare the aforementioned variables at the same time-point and resulting in a significant variation in the sampling date. In contrast, the G5D group had less variation because they visited the hospital regularly for hemodialysis. Moreover, regarding the measurement of follow up antibody titers 6 months after the second vaccination, since CKD stage progression was observed in some patients in the G4 and G5 groups, we could not compare antibody titers. However, antibody titers tended to be lower in the G5D group than in the G4 and G5 groups despite the shorter time since vaccination, and we observed a decrease in antibody titers after 6 months.

## Conclusions

We highlight two major factors of variability in vaccine response. First, in elderly patients with late-stage CKD, antibody titers tended to be lower in the G5D group than in the G4 and G5 groups despite the shorter time since vaccination; therefore, CKD stage progression might cause a decline in antibody titers. Second, waning immune responses were observed 6 months after administering the second dose in HD patients advocating a potential need for a third-dose booster vaccine after 6 months.

Although no threshold has been established for protective immunity, a third-dose booster COVID-19 vaccine after 6 months may be necessary to sustain a protective immunity, particularly in patients with late-stage CKD with a low immune response.

## Data Availability

All data generated or analyzed during this study are included in this published article.

## Abbreviations

CKD: Chronic kidney disease
RRT: Renal replacement therapy
